# Hsp70 Chaperones and Type I PRMTs Are Sequestered at Intranuclear Inclusions Caused by Polyalanine Expansions in PABPN1

**DOI:** 10.1371/journal.pone.0006418

**Published:** 2009-07-29

**Authors:** João Paulo Tavanez, Rocio Bengoechea, Maria T. Berciano, Miguel Lafarga, Maria Carmo-Fonseca, Francisco J. Enguita

**Affiliations:** 1 Instituto de Medicina Molecular, Faculdade de Medicina, Universidade de Lisboa, Lisboa, Portugal; 2 Department of Anatomy and Cell Biology, and “Centro de Investigación Biomédica en Red sobre Enfermedades Neurodegenerativas (CIBERNED)”, University of Cantabria, Santander, Spain; Auburn University, United States of America

## Abstract

Genomic instability at loci with tandem arrays of simple repeats is the cause for many neurological, neurodegenerative and neuromuscular diseases. When located in coding regions, disease-associated expansions of trinucleotide repeats are translated into homopolymeric amino acid stretches of glutamine or alanine. Polyalanine expansions in the poly(A)-binding protein nuclear 1 (PABPN1) gene causes oculopharyngeal muscular dystrophy (OPMD). To gain novel insight into the molecular pathophysiology of OPMD, we studied the interaction of cellular proteins with normal and expanded PABPN1. Pull-down assays show that heat shock proteins including Hsp70, and type I arginine methyl transferases (PRMT1 and PRMT3) associate preferentially with expanded PABPN1. Immunofluorescence microscopy further reveals accumulation of these proteins at intranuclear inclusions in muscle from OPMD patients. Recombinant PABPN1 with expanded polyalanine stretches binds Hsp70 with higher affinity, and data from molecular simulations suggest that expansions of the PABPN1 polyalanine tract result in transition from a disordered, flexible conformation to a stable helical secondary structure. Taken together, our results suggest that the pathological mutation in the PABPN1 gene alters the protein conformation and induces a preferential interaction with type I PRMTs and Hsp70 chaperones. This in turn causes sequestration in intranuclear inclusions, possibly leading to a progressive cellular defect in arginine methylation and chaperone activity.

## Introduction

Tandem arrays of simple repeats such as mono-, tri-, and tetranucleotides are common in eukaryotic genomes, and repeat instability is the cause for more than 40 neurological, neurodegenerative and neuromuscular diseases [1]. Repeat instability is a dynamic form of mutation that is most likely associated with DNA replication, repair and recombination. Intriguingly, there is an evolutionary trend towards longer trinucleotide repeats in humans relative to other species [2]. The majority of repeat-associated disorders are caused by expansions of trinucleotide repeats located in either coding or non-coding regions of the genome. While noncoding repeats may induce the generation of chromosome fragility, the silencing of the genes in which they are located, the modulation of transcription and translation, and the sequestering of proteins involved in cellular processes, repeats in the coding sequence can result in the generation of toxic or malfunctioning proteins. Disease-associated expansions of coding DNA triplets are translated into homopolymeric amino acid stretches of glutamine or alanine [3], [4].

Approximately 500 human proteins are predicted to contain polyalanine tracts, and disease-causing expanded alanine stretches have been identified in nine of these proteins [4]–[6]. With a single exception, all these proteins are transcription factors that play important roles during development. The expansion mutations in these transcription factors lead to a variety of symptoms including mental retardation and malformations. The exception is poly(A)-binding protein nuclear 1, PABPN1 (previously called PABP2), a protein involved in polyadenylation of mRNA precursors [7]. Polyalanine expansion in PABPN1 causes oculopharyngeal muscular dystrophy, OPMD [8]. OPMD is caused by expansions in a 6 GCG trinucleotide repeat tract ([GCG]_6_) located in the first exon of the PABPN1 encoding gene [9]. In the vast majority of patients the disease is inherited with heterozygous mutation carriers displaying alleles in the range from 2 to 7 additional GCG repeats [(GCG)_8–13_] [9].

Given the frequency of polyalanine stretches, their strong evolutionary conservation, and the deleterious effects of their expansion, it is likely that alanine tracts play an important role in protein structure and function. Recent in vitro and in vivo data suggest that expansions of polyalanine tracts beyond a certain threshold result in protein misfolding and aggregation [4].

Here we show that the normal polyalanine stretch in PABPN1 is predicted to be intrinsically unstructured and highly flexible, whereas peptides corresponding to the extended PABPN1 tend to form a helical secondary structure. We further show that expansions of the polyalanine tract result in increased association with Hsp70 chaperones and type I arginine methyl transferases. These findings raise the possibility that sequestration by expanded PABPN1 may cause a progressive cellular defect in both protein modifications by arginine methylation and chaperone activity.

## Results

### Identification of proteins that associate preferentially with expanded PABPN1

**Figure 1:**
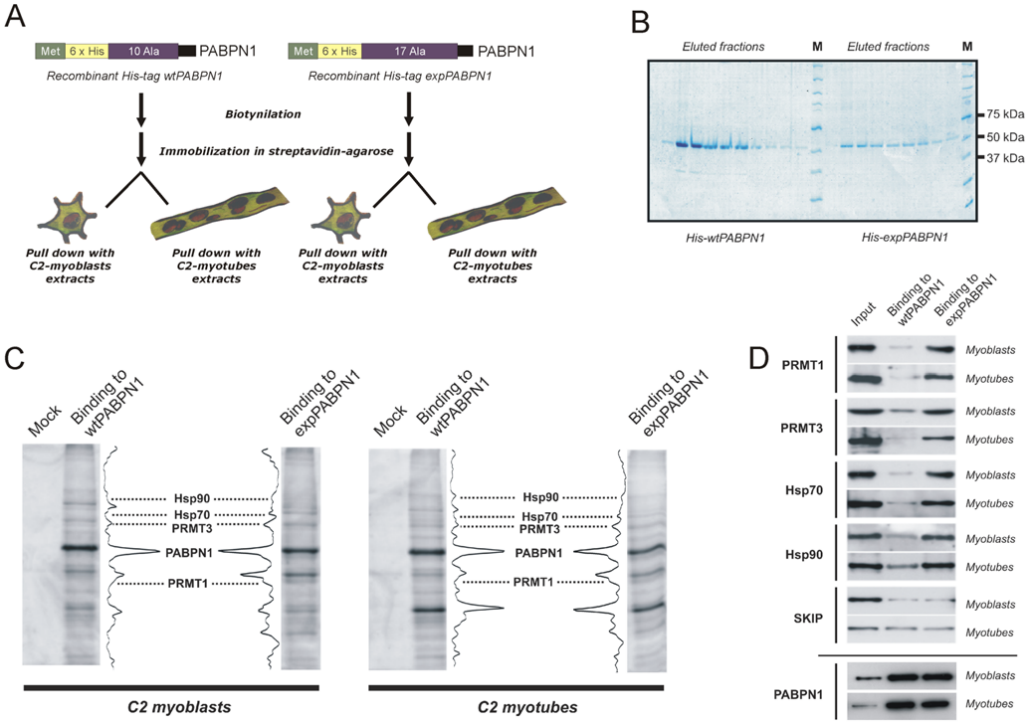
Identification of proteins that associate preferentially with expanded PABPN1. (A) Purification strategy. Recombinant, His-tagged PABPN1 containing either a normal homopolymer of 10 alanine residues or an expanded tract of 17 alanine residues was expressed in baculovirus system and purified by nickel affinity. Normal and expanded PABPN1 were then biotinylated, immobilized to streptavidin-agarose beads and incubated with RNase-treated extracts from undifferentiated C2 (myoblasts) and differentiated C2 (myotubes) cells. (B) Coomassie-stained SDS polyacrylamide gel of purified recombinant HIS-PABPN1 used for pull-down experiments. (C) Bound proteins were eluted, separated by 10% SDS-PAGE, and detected by silver staining. The gel bands that stained with higher intensity in the lanes corresponding to expanded PABPN1 were excised and proteins identified by mass spectrometry. The identity of the bands is indicated. As a control, the same amount of extract was incubated with beads devoid of any immobilized protein (control lanes). (D) Proteins bound to normal and expanded PABPN1 were eluted, separated by 10% SDS-PAGE, blotted to nitrocellulose, and probed with the antibodies against the indicated proteins. Total protein from C2 cell extract (input) was run in parallel. Lower panel shows the corresponding loading control for wt- and expanded PABPN1 proteins in cell free extracts obtained from myoblasts and myotubes.

In order to examine if polyalanine tract expansion affects the binding of cellular proteins to PABPN1, we performed pull-down experiments from cell extracts with immobilized PABPN1 variants (Fig. 1). For this purpose, normal and expanded human PABPN1 was expressed in baculovirus system, purified and biotinylated. Then, the biotinylated PABPN1 was immobilized on streptavidin-agarose beads and incubated with extracts from undifferentiated and differentiated C2 cells (Fig. 1A, B). Cell extracts were pre-treated with RNase A to avoid detection of protein interactions mediated by RNA. Cellular proteins that bound to the beads were eluted and analyzed by SDS-PAGE. The gel bands that stained with different intensities in the lanes corresponding to normal and expanded PABPN1 were excised and proteins identified by mass spectrometry (Fig. 1C). The results are summarized in Table 1, which lists the number of unique peptides identified for each protein in the different experiments.

**Table 1:** Mass spectrometry analysis of PABPN1-interacting proteins.

NAME	MW	ACCESSION#	PEPTIDES			
			Myoblasts		Myotubes	
			wt	exp	wt	exp
PRMT1	39	Q8C2D7	5	18	4	17
PRMT3	59	Q922H1	6	14	3	10
HSP70 1A	70	Q61696	3	21	5	19
HSP70 1B	70	P17879	2	15	6	14
Hsc73	70	JC4853	4	11	3	9
HSP70 1L	70	I49761	1	5	2	8
HSP90 alpha	84	P07901	4	11	6	16
HSP90 beta	83	P11499	4	10	7	19
Laminin receptor 1	32	Q8BNL2	1	1	2	1
CREB-binding protein	264	Q8QZV8	1	1	0	0
N-RAP	133	T37192	0	0	2	1
YBOX1	35	P62960	3	4	4	3
MYB-1 A	36	Q60950	2	5	6	4
MYB-1 B	35	Q60951	2	2	4	3
EF2	95	P58252	5	4	2	3
DDEF2	106	Q7SIG6	1	1	3	2
EF1A1	50	P10126	7	9	3	3
Gspt1	49	Q8BPH0	0	1	1	1
hnRNP U	87	O88568	9	8	7	8
IMPORTIN 7	119	Q9EPL8	3	3	2	1
PABPN1	32	Q8CCS6	6	5	6	6
SkiP	61	Q9CSN1	3	4	7	8

The number of unique peptides identified associated with normal (wt) PABPN1 is compared with the number of unique peptides identified associated with expanded (exp) PABPN1 in undifferentiated (myoblasts) and differentiated (myotubes) C2 cells. Accession # indicates UniProt accession numbers. MW indicates the calculated molecular weight in kilodaltons.

Immunoblot analysis confirmed that PRMT1, PRMT3, Hsp70 and Hsp90 are more abundantly associated with expanded PABPN1 relative to normal PABPN1, and similar results are observed in undifferentiated and differentiated C2 cells (Fig. 1D). In contrast, similar levels of Ski-interacting protein (SKIP), which was previously identified as a PABPN1 interactor [10], are detected in association with normal and expanded PABPN1. However, the interaction between SKIP and PABPN1 is enhanced in differentiated C2 cells compared to undifferentiated cells, consistent with the view that SKIP co-operates with PABPN1 to stimulate myogenesis [10].

### PRMT1 and PRMT3 are present in OPMD intranuclear inclusions

**Figure 2:**
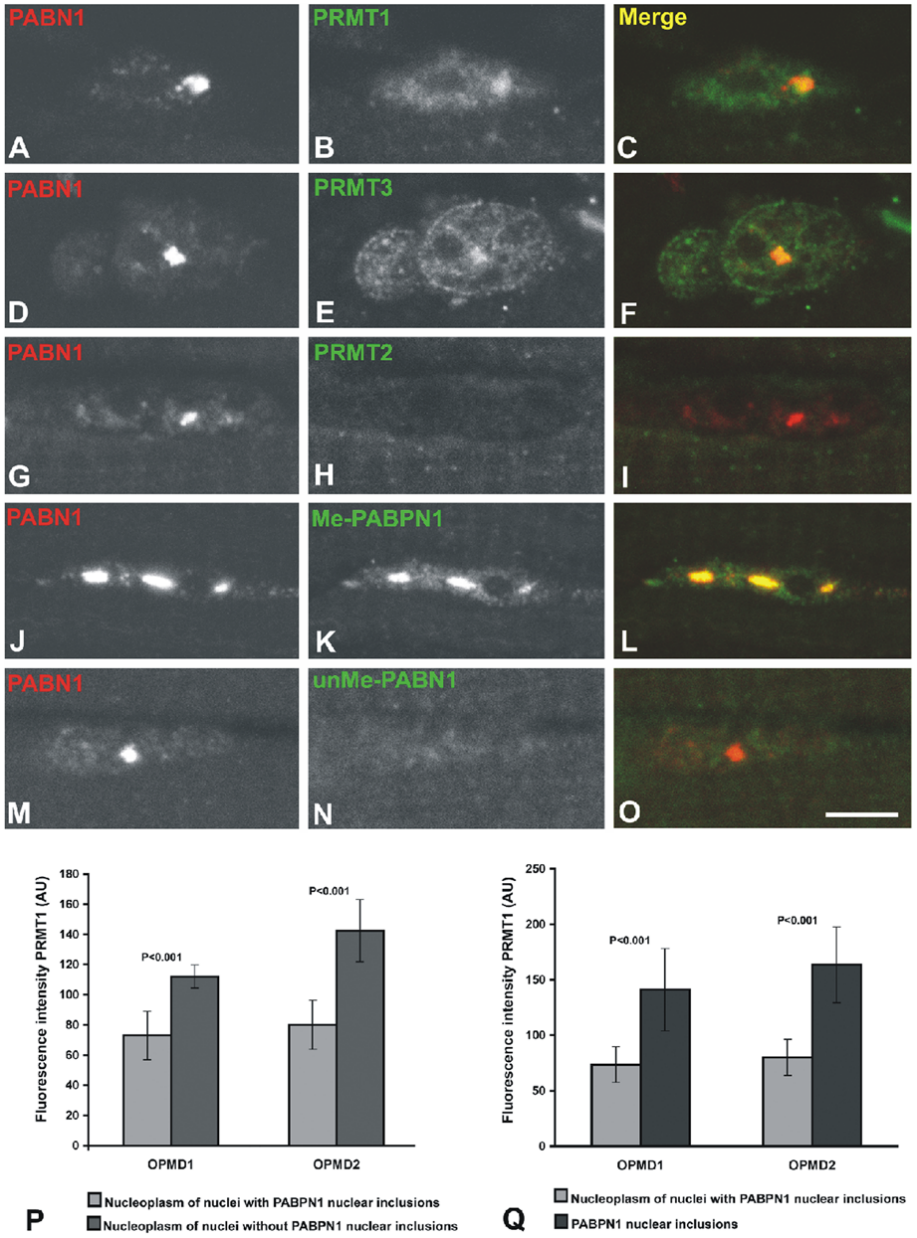
PRMT1 and PRMT3 accumulate in OPMD intranuclear inclusions. (A–O) Immunofluorescence was performed on biopsy samples of quadriceps muscle from two OPMD patients using the indicated antibodies. Scale bar = 5 µm. (P–Q) From each patient (OPMD1 and OPMD2), approximately 60 nuclei labelled with anti-PRMT1 antibody were analyzed. The mean fluorescence intensity measured in the nucleoplasm is higher in nuclei devoid of inclusions compared to nuclei with inclusions (P). The mean fluorescence intensity measured in the nuclear inclusions is approximately two-fold higher than the intensity measured in the nucleoplasm of nuclei with inclusions (Q). Quantitative measurements of fluorescence intensity were performed with Image J software and expressed in arbitrary units (AU). Results are presented as mean fluorescence intensity±SE; *p* values (Student's *t*-test) are indicated.

As the major pathological hallmark of OPMD consists of intranuclear inclusions formed by deposition of PABPN1 fibrils, we next analyzed the sub-cellular distribution of proteins identified as preferentially associated with expanded PABPN1. Immunofluorescence was performed on muscle fibers from two OPMD patients that have one normal *PABPN1* allele (GCG_6_) and one expanded allele (GCG_11_) coding for a protein with a polyalanine tract of 15 residues. Typical intranuclear inclusions were detected using anti-PABPN1 antibodies (Fig. 2A, D, G, J, M), and double-labelling experiments reveal that both PRMT1 (Fig. 2B, C) and PRMT3 (Fig. E, F) are concentrated in the inclusions. In contrast to PRMT1 and PRMT3, which are protein arginine methyltransferases involved in the modification of PABPN1 [11], [12], PRMT2, which does not methylate PABPN1, is not detected in the inclusions (Fig. 2H, I). The intranuclear inclusions are intensely stained by anti-peptide antibodies that specifically recognize asymmetrically dimethylated PABPN1 (Fig. 2K, L), but do not react with antibodies specific to unmethylated PABPN1 (Fig. 2N, O). These results indicate that pathological aggregates of PABPN1 consist of methylated protein and accumulate PRMT1 and PRMT3.

Taking into account the biochemical evidence indicating that PRMT1 and PRMT3 bind preferentially to expanded PABPN1, one possibility is that these enzymes become sequestered in the pathological PABPN1 aggregates. To address this view, we estimated the fluorescence intensity corresponding to the sub-nuclear distribution of PRMT1. The proportion of visible intranuclear inclusions that stained positive for PRMT1 was 48% (n = 60) and 41% (n = 58) in patient #1 and patient #2, respectively. In both patients, the intensity of PRMT1 fluorescence was approximately two fold higher in the inclusions than in the surrounding nucleoplasm (Fig. 2Q). Consistent with the sequestration hypothesis, the intensity of PRMT1 fluorescence was significantly higher in the nucleoplasm of nuclei that do not contain visible inclusions than in the nucleoplasm of nuclei with inclusions (Fig. 2P).

### Hsp70 accumulates in OPMD intranuclear inclusions and binds with higher affinity to expanded PABPN1 *in vitro*

**Figure 3:**
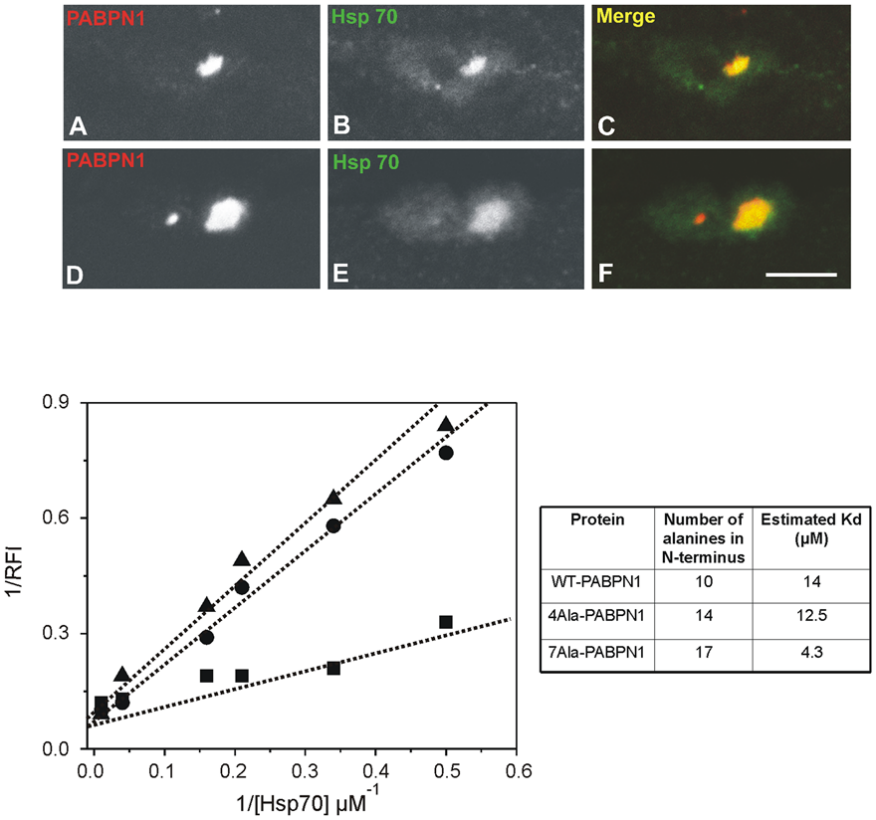
Hsp70 accumulates in OPMD intranuclear inclusions and binds with higher affinity to expanded PABPN1 *in vitro*. (A–F) Immunofluorescence was performed on biopsy samples of quadriceps muscle from two OPMD patients using the indicated antibodies. Scale bar = 5 µm. Tryptophan emission fluorescence spectra were recorded in response to the addition of increasing amounts of purified recombinant Hsp70 to purified recombinant normal (wt) and expanded PABPN1 variants. The graph depicts a double reciprocal plot of the increase of fluorescence intensity at the maximum wavelength of PABPN1 variants in response to increasing concentrations of Hsp70. Concentration of PABPN1 variants was 100 µM. (▴) wt-PABPN1, (•) 4Ala-PABPN1 and (▪) 7Ala-PABPN1. RFI, relative fluorescence intensity. The estimated dissociation constants (Kd) for each complex are indicated in the table.

Having established that PRMT1 and PRMT3 associate preferentially with expanded PABPN1 and concentrate in OPMD intranuclear inclusions, we next examined the sub-cellular distribution of Hsp70 and Hsp90 in muscle fibers from OPMD patients. Immunofluorescence analysis revealed a clear concentration of Hsp70 in the PABPN1 inclusions (Fig. 3), while antibodies anti-Hsp90 did not stain these structures (not shown). To further characterize the influence of polyalanine extension on the interaction between Hsp70 and PABPN1, we performed steady-state tryptophan fluorescence spectroscopy. PABPN1 has a single tryptophan residue located in the C-terminal region that can be used to monitor changes in the amino acid environment caused by protein-protein interactions. Tryptophan emission fluorescence spectra were recorded in response to the addition of increasing amounts of purified recombinant Hsp70 to purified recombinant normal and expanded PABPN1 variants. Contribution of Hsp70 to the fluorescence spectra was subtracted in all the cases. For each PABPN1/Hsp70 complex, the relative dissociation constant (Kd) was calculated (Fig. 3). The results show that, compared to normal PABPN1, the interaction with Hsp70 is stronger for the expanded protein with 14 alanines and even stronger for the expanded variant with 17 alanines (Fig. 3). This suggests that the number of residues in the polyalanine tract of PABPN1 correlates with the binding affinity to Hsp70.

### The polyalanine stretch is part of an intrinsically unstructured segment within PABPN1

**Figure 4:**
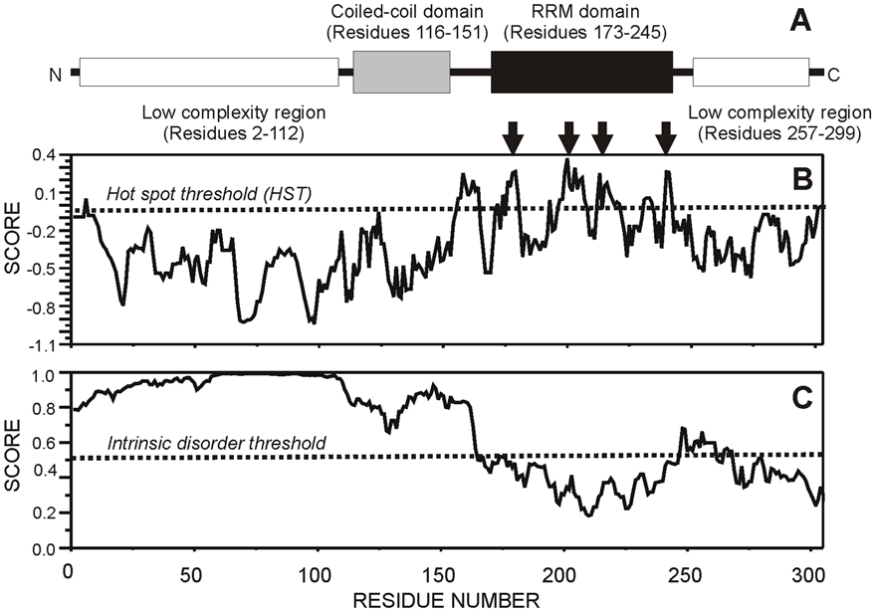
Computational analysis of PABPN1 structure. (A) Domains and structural elements in PABPN1 protein sequence. (B) Analysis of the aggregation propensity performed by AGGRESCAN server [15]. Hot-spots determined by the server are marked with arrows. (C) Prediction of intrinsically disordered segments in the protein using the IUpred server [19]. In panels B and C the threshold for significant scores is defined by a dotted line.

PABPN1 is a 306 amino acid protein and its primary structure suggests a separation into four domains: an N-terminal segment encompassing amino acids 2–112; a coiled-coil domain corresponding to amino acids 116 to 151 [13], [14]; a single RNA recognition motif (RRM) corresponding to amino acids 173–245 and a C-terminal domain of approximately 49 amino acids (257–306) (Fig. 4A). In order to bioinformatically analyze the presence of “hot-spots” for protein aggregation within PABPN1 amino acid sequence we used the AGGRESCAN server [15]. The algorithm uses biological data from already known samples of aggregating polypeptides to compute a score for the aggregation propensity of a short amino acid region in a protein sequence. The results obtained with normal PABPN1 sequence show that higher scores for aggregation propensity are located within the RRM domain (Fig. 4B). This is consistent with our previous findings indicating that normal PABPN1 is inherently aggregation-prone when exogenously expressed and that mutations in the RRM prevent aggregate formation [16]. The low score for aggregation propensity detected in the N-terminal region (Fig. 4B) is also in agreement with our observation that aggregation of exogenously expressed PABPN1 is independent from the polyalanine stretch [16]. In further agreement with this view, two recent studies reported the structure of the RRM domain of nuclear poly(A)-binding proteins and showed that this domain mediates formation of protein dimers [17], [18]. The region involved in interaction between each protein subunit corresponds to higher scores in the aggregation propensity profile (Fig. 4B, arrows).

To further examine the structural features of the N-terminal segment of PABPN1, we used the IUpred server [19]. The results reveal that this region of the protein is predicted not to fold into well-defined, ordered tertiary structures but to correspond to an intrinsically disordered segment (Fig. 4C). The region has a combination of low overall hydrophobicity and low sequence complexity that leads to propensity to be disordered [20], [21]. In fact, with the exception of the RRM domain, most of PABPN1 structure is predicted as unfolded or lacking a tertiary structure under physiological conditions (Fig. 4C). Intrinsic disorder is a unique feature that allows proteins to be very dynamic in their structure and to participate in several pathways at the same time through binding to multiple partners via high-specificity and low-affinity interactions play an important role [22], [23].

### Polyalanine expansions are coupled with disorder-to-order transitions

**Figure 5:**
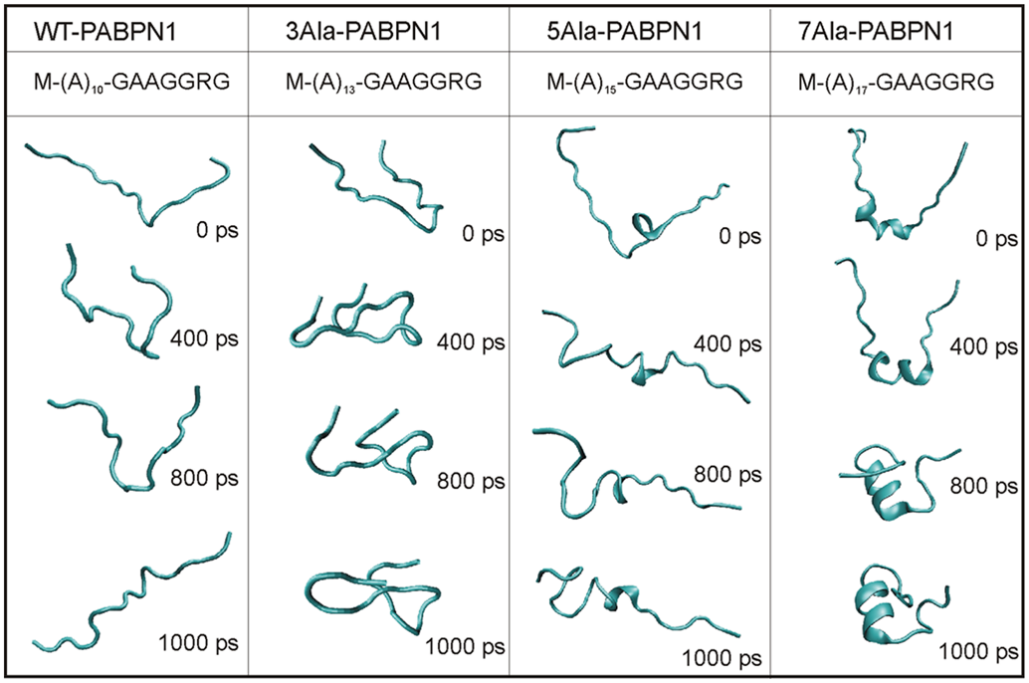
Molecular dynamics snapshots of normal and expanded PABPN1 N-terminal peptides. Conformational behavior of peptides corresponding to the N-terminal segment of PABPN1 variants were studied by molecular dynamics simulation during a time scale of 1000 ps. The figure shows selected snapshots along the molecular dynamics simulation trajectory for wild-type (wt) PABPN1 and expanded variants with 3, 5 and 7 additional alanines. Sequences of the peptides are indicated at the top of the table. Figure prepared with PyMol [52].

**Figure 6:**
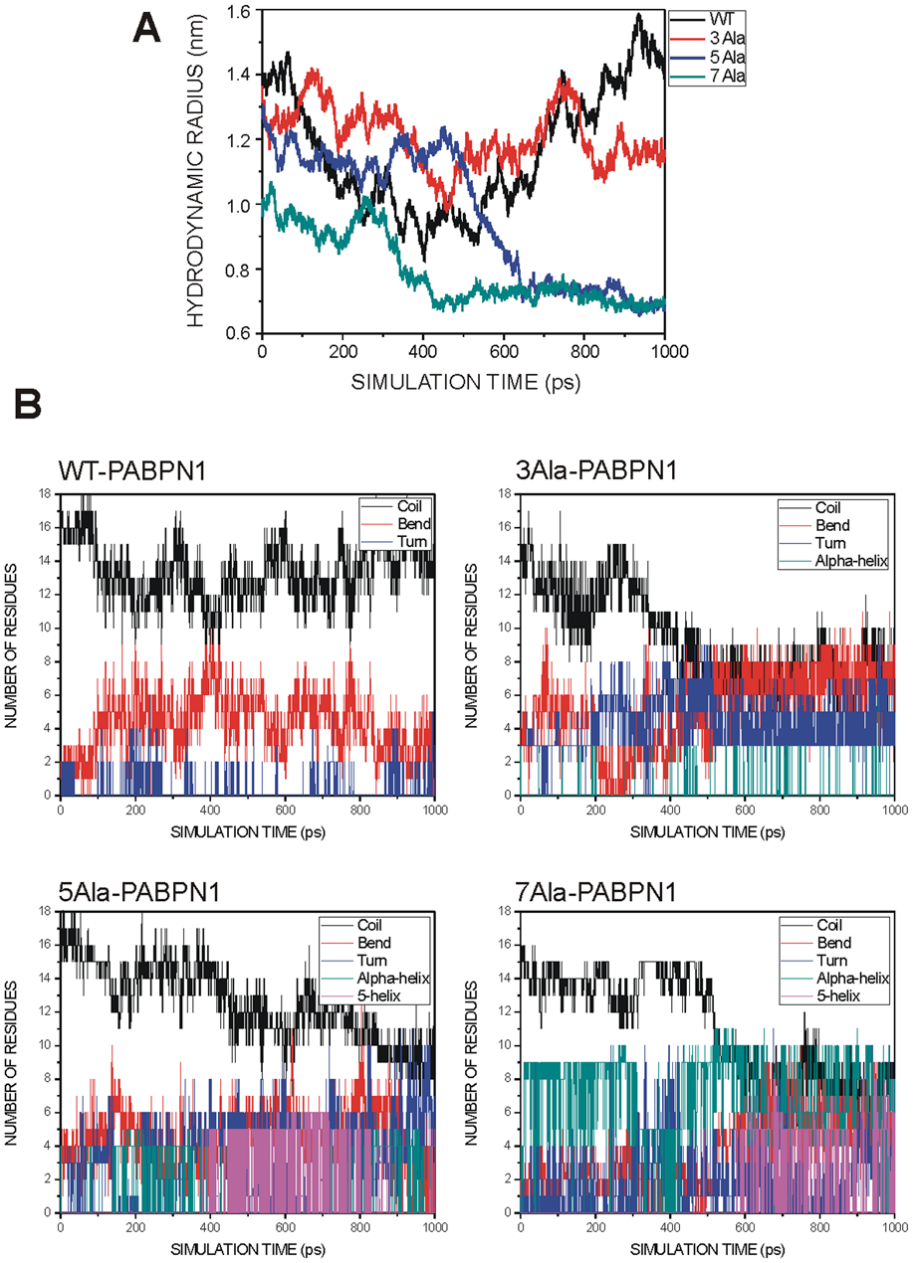
Conformational characteristics of normal and expanded PABPN1 N-terminal peptides. (A) Evolution of the hydrodynamic radius along the molecular dynamics simulation trajectory for wild-type (wt) PABPN1 and expanded variants. (B) Evolution of secondary structure elements along the molecular dynamics trajectories performed by DSSP [53], [54] for wild-type (wt) PABPN1 and expanded variants. Each type of secondary structure element is represented in a different color. The number of amino acids belonging to each secondary structure type is represented versus the simulation time.

Next, we asked how an expansion in alanine residues may affect the natively disordered structure of the N-terminal segment of PABPN1 using molecular dynamics simulations. N-terminal peptides from wtPABPN1 and extended variants, containing 10, 13, 15 and 17 alanine residues, were studied during a time scale of 1000 ps. Figure 5 shows several representative snapshots of the molecular dynamics trajectory for each peptide. These snapshots were selected to demonstrate typical changes of the peptide structure along the simulation. The simulation results reveal that 10-alanine peptides are very flexible, with no preference for any stable secondary structure. As the number of alanines increases there is an increment of structured secondary elements along the molecular dynamics trajectory. The most striking difference is observed with the 15- and 17-alanine peptides, which form a short alpha helical structure. Analyzing the evolution of the hydrodynamic radius during the simulation shows that, in contrast to peptides with 10 or 13 alanines, peptides with 15 or 17 alanines rapidly evolve to a very compact structure characterized by an abrupt decrease of the hydrodynamic radius of the molecule (Fig. 6A). Analysis of the evolution of secondary structure as a function of time in the simulation was determined by analysis of the trajectories with GROMACS [24]. The results show that 10-alanine peptides behave as a very flexible ensemble along the whole simulation with the majority of the residues in coiled structure (Fig. 6). The simulation with 13-alanine peptides starts with a high content of amino acids in coiled conformation, but at 400 ps the system evolved in a different way, with a decrease of the contribution of the coiled structure and an increase in the residues present in bended fragments. This suggests the presence of a more compact conformation but without any predominant secondary structure elements. The simulation with either 15- or 17-alanine peptides starts with a high content of flexible, non-structured regions and evolves to a structured segment mainly constituted by a helical secondary structure. Particularly interesting is the presence of a stable helical fragment in the 17-alanine peptide at the end of the simulation. This helical fragment is composed of 14 residues distributed by a main stretch of 8 amino acids in α-helix structure and also by a small segment of 5 amino acids that forms a typical 5α-helix (Fig. 6B). The 5α-helix is a subtype of helical secondary structure in which each amino acid corresponds to a 87° turn in the helix (i.e., the helix has 4.1 residues per turn), and a translation of 1.15 Å along the helical axis, being more expanded and flexible than a common α-helix [25]. In conclusion, data from molecular simulations suggest that expansions of polyalanine tracts result in transition from a disordered, flexible conformation to a stable helical secondary structure.

### Polyalanine expansion associates with increased protein hydrophobicity

**Figure 7:**
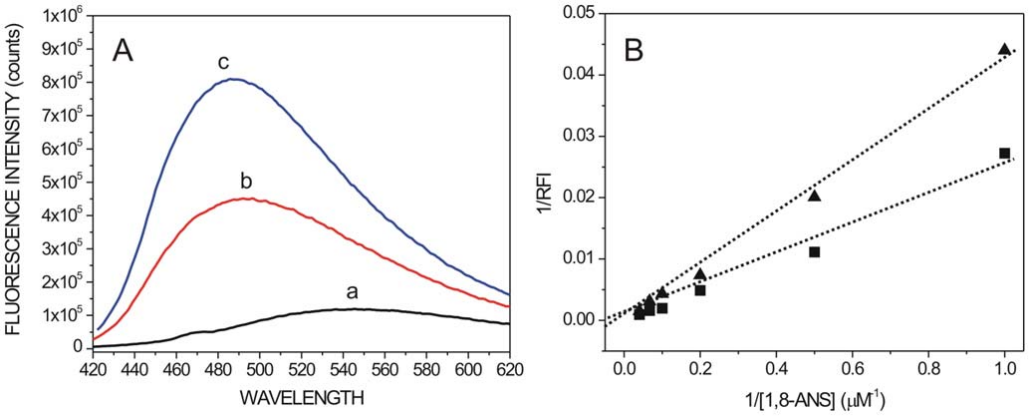
Effect of alanine expansion on exposure of PABPN1 hydrophobic domains. (A) The degree of exposure of hydrophobic domains was determined by 1,8-ANS fluorescence analysis. Fluorescence emission spectra of 1,8-ANS (5 µM) either free (a) or bound to 5 µM recombinant wt-PABPN1 (b) and 5 µM recombinant 7Ala-PABPN1 (c). (B) Determination of the dissociation constants (Kd) for the complexes between 1,8-ANS and PABPN1 variants. (▴) wt-PABPN1 and (▪) 7Ala-PABPN1 RFI, relative fluorescence intensity.

The predicted conformational transitions caused by alanine expansions are expected to increase hydrophobicity of mutant PABPN1 proteins, thereby contributing to the increased affinity for Hsp70 binding. To experimentally test this view, we measured the exposure of hydrophobic domains in normal (wt) and expanded (7Ala) PABPN1 by means of 1-Anilino naphthalene-8-sulfonic acid (1,8-ANS) fluorescence analysis. 1,8-ANS is a fluorescent probe that exhibits an increased and blue-shifted emission upon binding to exposed hydrophobic patches [26]. Analysis of the emission spectrum of 1,8-ANS bound to normal and expanded PABPN1 is shown in Figure 7. Consistent with the predicted abundant intrinsic unstructured regions in PABPN1 (Fig. 4), the position of the maximum of emission (λ_max_) obtained for the complexes between 1,8-ANS and wt- or 7Ala-PABPN is red-shifted in relationship to the range (about 468–477 nm) usually reported for globular proteins with a stable tertiary structure [27]. Noteworthy, λ_max_ for 1,8-ANS bound to 7Ala-PABPN1 (485 nm) showed a blue-shift compared with λ_max_ for 1,8-ANS bound to wt-PABPN1 (493 nm). Moreover, binding of 1,8-ANS to 7Ala-PABPN1 resulted in increased emission intensity compared with binding of 1,8-ANS to wt-PABPN1 (Fig. 7A). Estimation of dissociation constants from fluorescence spectroscopy data (Fig. 7B) revealed that 1,8-ANS binds to expanded PABPN1 with approximately two-fold more affinity (Kd = 42 µM) than it binds to normal PABPN1 (Kd = 75 µM). Taken together these results show that compared to normal PABPN1, the mutant protein contains an increased amount of exposed hydrophobic patches to the surface of the protein.

## Discussion

Using a biochemical approach, we have identified two classes of proteins that bind differentially to PABPN1 containing either normal or expanded polyalanine tracts. One class comprises protein arginine methyl transferases 1 and 3 (PRMT1 and PRMT3), two enzymes responsible for methylation of arginine residues in the C-terminal domain of PABPN1 [11], [12], [28]. PRMT1 and PRMT3 belong to the so-called type I PRMTs, which catalyze asymmetrical dimethylation of arginine residues, whereas type II PRMTs catalyze symmetrical dimethylation [29]. Most substrates for type I enzymes bind RNA, including heterogeneous nuclear RNA binding proteins (hnRNPs), which collectively account for 65% of the nuclear asymmetric dimethylarginine [30]. PRMT1 is the predominant type I enzyme in tissues [31]. Murine embryos deficient in PRMT1 fail to develop, indicating a fundamental role for the protein, but *Prmt1* ^−/−^ cells are viable [32]. Consistent with some functional redundancy, PRMT3 and PRMT6 suffice to methylate PABPN1 in *Prmt1* ^−/−^ cells [12]. PRMTs have been implicated in a wide variety of cellular processes, but the biochemical and biological functions of asymmetrical arginine dimethylation remain largely unknown [29]. Here, we found that PRMT1 and PRMT3 associate preferentially with expanded PABPN1 and become accumulated at OPMD intranuclear inclusions. This raises the possibility that binding of methyltransferases to expanded PABPN1 accumulated in nuclear inclusions leads to a decreased availability of the enzymes in the nucleoplasm, which may in turn reduce the methylation status of other cellular targets. We therefore hypothesize that defects in asymmetrical arginine dimethylation secondary to sequestration of PRMT1 and PRMT3 in nuclear inclusions may contribute to the pathophysiology of OPMD.

Heat shock proteins represent another class of proteins that we found preferentially associated with expanded PABPN1 and concentrated at OPMD intranuclear inclusions. Hsp70 molecular chaperones play diverse roles in cells and all such functions are mediated by interaction of extended, hydrophobic regions of substrate proteins with the Hsp70 C-terminal substrate-binding domain [33]. Here we show biochemically that Hsp70 binds with higher affinity to expanded PABPN1 *in vitro*, and that binding affinity is proportional to the length of the polyalanine stretch. We further show that the normal polyalanine stretch in PABPN1 is predictably intrinsically unstructured and highly flexible, whereas alanine expansion causes a disorder-to-order conformational transition. A major change involving formation of a stable helical secondary structure is predicted to take place when the polyalanine stretch expands to 15 or 17 residues. By increasing hydrophobicity, as confirmed by 1,8-ANS-fluorescence analysis, these changes most likely contribute to the increased affinity for Hsp70 binding.

Several neurodegenerative disorders including Alzheimer, Parkinson and Huntington diseases, amyotrophic lateral sclerosis and spinocerebellar ataxias are associated with the formation of non-native protein structures that have been described as oligomers, aggregates, plaques or inclusions. These are thought to result from the combined expression of misfolded proteins and disruption of protein folding quality control [34]. Molecular chaperones such as Hsp70 are detected in disease-related non-native protein structures, and it has been suggested that this interaction reflects the efforts of Hsp70 to dissociate the aggregates [35]. In this study we provide novel evidence supporting this view. Our results suggest that Hsp70 molecular chaperones become trapped by aggregates of expanded PABPN1 in muscle cell nuclei. Possibly, recruitment of Hsp70 chaperones to expanded PABPN1 reflects an effort to prevent aggregate formation and/or to dissociate the protein from aggregates. However, as this protective effort fails and larger nuclear inclusions accumulate in the nucleus, a progressively larger proportion of molecular chaperones may be sequestered in the PABPN1 aggregates, thus decreasing the proportion of chaperones available to perform other essential functions in the cell. This would aggravate the diminished capacity of heat shock gene induction that seems to occur later in life [34].

In conclusion, this study highlights two novel mechanisms that may contribute to OPMD. By interacting preferentially with expanded PABPN1, type I PRMTs and Hsp70 chaperones are sequestered in pathological nuclear inclusions. Consequently, cells may become progressively defective in asymmetrical arginine dimethylation activity and in heat shock response. Defects caused by impaired type I PRMT activity could be expected at the level of signal transduction pathways to regulate gene expression and cell proliferation, whereas diminished chaperone activity could impinge on protein translation, import of proteins into organelles, response to stress and protein folding quality control.

Our data further reveal that the normal polyalanine stretch in PABPN1 is predicted to be intrinsically unstructured and highly flexible. In contrast, peptides corresponding to the extended PABPN1 variants N-terminal regions and containing 15 or 17 alanine residues tend to form a helical secondary structure. The polyalanine expansion in the N-terminal peptide of PABPN1 induces the formation of an alpha-helical segment proportional in size to the number of alanines in expansion. This ordered alpha helical segment is located within a predicted unfolded protein section that comprises the first 112 amino acids, and that has been involved in PABPN1 interaction with other proteins [36], [37]. Interestingly, molecular dynamics data also showed the presence of a 5α-helix (also known as π-helix) in the 7Ala-expanded PABPN1 N-terminal peptide simulations. This π-helices are more expanded and higher-entropy structures than a classical α-helix and have been involved in certain specific protein-protein interactions [25]. The presence of this helical-segment in expanded variants of PABPN1 could modulate their interactions properties by changing partner specificity and/or affinity.

We show that Hsp70 binds with higher affinity to pathologically expanded PABPN1 and associates with intranuclear inclusions characteristic of OPMD muscle fibers. Based on data analysis from molecular simulations we propose that expansions of the PABPN1 polyalanine tract beyond a certain threshold result in transition from a disordered, flexible conformation to a stable helical secondary structure that enhances the binding to Hsp70. It is well known that molecular chaperones are cellular helpers for protein folding. Specifically, Hsp proteins are known to interact with nascent polypeptides and hydrophobic protein chains to promote their folding [38]. In OPMD, expanded polyalanine tracts in PABPN1 could act as a signal to promote a misfolding response. This could be due to the presence of a hydrophobic and structured alpha-helical fragment (constituted by the expanded polyalanine stretch) within a hydrophilic N-terminal region. This signal could recruit specific chaperones, mainly belonging to the Hsp family, and cause a misbalance in the levels of these molecules that could be accumulative on time. Furthermore, the formation of this stable alpha helix in the N-terminal region of PABPN1 due to the pathological expansion could be also responsible for the misregulation in the binding specificity of PABPN1 to some of its physiological partners. Indeed, it is well known that protruding alpha helices and more in detail polyalanine stretches are abundant in higher eukaryotes and they are proposed to be involved in regulatory networks mediating protein-protein interactions [39].

Although the aggregative behavior of expanded PABPN1 protein has been proposed as the cause of OPMD, a propensity to aggregate is not exclusive of the expanded protein and can also be observed in normal PABPN1 [16], [40]-[43]. Based on the evidence reported here, we propose that polyalanine tract expansions alter the protein conformation and change the binding properties of interacting proteins independently of the formation of intranuclear inclusions. Together with protein aggregation, this likely represents an additional mechanism contributing to the disease. Indeed, evidence that mutant proteins can induce neurodegeneration by an apoptotic mechanism in the absence of nuclear inclusions was first reported in a cellular model of Huntington's disease [44]. The concept that proteotoxicity of expanded PABPN1 is caused by altered protein networking has important implications for the development of novel therapeutic strategies, such as druggable modifiers of altered protein function [45].

## Materials and Methods

### Pull-down assays with biotinylated proteins

The normal and expanded forms of recombinant PABPN1 purified from insect cells (see supplementary material S1 for details) were biotinylated using EZ-Link PEO-Maleimide Activated Biotin (Pierce), according to the manufacturer's instructions. Buffer exchange to remove unincorporated PEO-biotin was performed using NAP-5 or NAP-10 columns (Pharmacia Biotech). For each binding reaction, 15 µl of streptavidin-agarose beads (Sigma-Aldrich) was presaturated with biotinylated PABPN1 (2 µg) diluted in 20 mM Tris, pH 8.0, 200 mM KCl and 10% glycerol. The beads were washed three times with 20 mM Tris, pH 8.0, 200 mM KCl, 10% glycerol, and incubated in the same buffer supplemented with 100 µl of C2 cell extract in a total volume of 150 µl for 4 h at 4°C. The beads were then washed three times with 20 mM Tris, pH 8.0, 200 mM KCl, 10% glycerol, followed by three further washes with 50 mM Tris, pH 7.5, 150 mM potassium acetate, and 2 mM MgCl2, and a final wash in 50 mM Tris, pH 7.5. Bound proteins were eluted in 20 µl of SDS sample buffer and analyzed by SDS-PAGE and Western blotting.

### Cell culture and preparation of cell extracts

SF21 cells (an insect cell line highly susceptible to infection with baculovirus) were cultured in suspension in SF-900 II SFM medium (Invitrogen, Paisley, Scotland). C2 cells (a murine myogenic cell line derived from perinatal mouse skeletal muscle) were cultured as monolayer in Dulbecco's Modified Eagle's Medium (DMEM; Gibco Life Technologies, Rockville, MD) supplemented with 20% FCS. Cells were grown at 37°C in a humidified atmosphere containing 5% CO_2_ and maintained at sub-confluence to proliferate. To induce differentiation, C2 cells were plated at a density of 4×10^6^ cells per 35 mm culture dish and grown to confluence for 24 h. The medium was then changed to DMEM supplemented with 2% horse serum (GIBCO), and replaced every two days. Four days after culture in differentiation medium, C2 cells formed myotubes that stained positive for myosin heavy chain, a late marker of muscle differentiation.

Frozen C2 cell pellets were resuspended and incubated in 20 mM Tris pH 8.0, 500 mM NaCl, 0.1% NP40, 0.1% Deoxycholate, 0.1% Triton X-100 and protease inhibitors, for 1 h at 4°C. The extracts were clarified by centrifugation for 45 min at 14000 rpm, and then digested with 0.5 µg/µl RNase A (Sigma-Aldrich), for 1 hour at 37°C. After RNase treatment, extracts were dialyzed to 20 mM Tris pH 8.0, 200 mM KCl and 10% glycerol, for 5 h, and then cleared by centrifugation.

### Protein Identification by Mass-Spectrometry

Cellular proteins that bound to biotinylated PABPN1 immobilized on streptavidin-agarose beads were eluted, separated by SDS/PAGE, and stained either with Coomassie blue or silver staining. The protein bands of interest were excised from the gel and were analyzed by matrix-assisted laser desorption/ionization MS (MALDI-MS) and identified in the National Center for Biotechnology Information non-redundant database by using MASCOT (Matrix Science, London) as a search engine.

### SDS-PAGE and immunoblotting

SDS-PAGE and Western blotting were performed as previously described [46]. Proteins were separated by SDS-PAGE on 10% polyacrylamide minigels (BioRad Laboratories, Richmond, California) and electroblotted to a nitrocelulose membrane. Membranes were washed in PBS, blocked with PBS 5% low fat milk for at least 1 h and incubated with specific primary antibodies diluted in PBS 2.5% low fat milk at 4°C overnight. Membranes were then washed 3×15 minutes in PBS-Tw 2.5% low fat milk, incubated with appropriated secondary antibodies conjugated with horseradish peroxidase (BioRad Laboratories, Richmond, California) and developed using a chemiluminescence reaction (ECL; Amersham Buchler GmbH, Braunschweig, Germany).

### Immunofluorescence

Immunofluorescence was performed on biopsy samples from vastus medialis (quadriceps) from two OPMD patients, after appropriate informed consent. Tissue samples were embedded in diethylene glycol and 1 mm thick sections were processed as previously described [47].

### Antibodies

The following primary antibodies were used in this study: rabbit polyclonal serum anti-PABPN1 [48]; mouse polyclonal serum raised against recombinant human PABPN1 [49]; rabbit anti-peptide antibodies recognizing asymmetrically methylated and unmethylated PABPN1 [12]; mouse monoclonal anti-SKIP antibody (Sigma Aldrich., St Louis, MO), rabbit polyclonal anti-Hsp70 antibody (Abcam and Stressgene) and rabbit polyclonal anti-Hsp 90 antibody (Abcam); rabbit polyclonal antibodies anti-PRMT1, anti-PRMT2 and anti-PRMT3 [12], [50].

### *In vitro* interactions of PABPN1 with Hsp70 by fluorescence spectroscopy

Fluorescence spectroscopy studies were performed with recombinant PABPN1 variants expressed and purified from *E.coli*, and with Hsp70 protein expressed and purified from *Pychia pastoris* (see supplementary material S1 for details) in a FS920 Steady-state fluorescence spectrophotometer from Edinburgh Instruments. The protein samples in interaction buffer (25 mM HEPES buffer, pH 7.5, 100 mM NaCl and 1 mM dithiothreitol) were gently stirred at 30°C, excited with a 285-nm light in a quartz cuvette and the emission spectra recorded (scan speed 1 nm/s and bandwidth 5 nm). All studies were made using steady-state fluorescence conditions and the fluorescence contribution of the solvent was subtracted in each sample.

Kinetic studies for determination of the affinity constants were carried out as described previously [51]. Assuming a simple association model between PABPN1 and Hsp70 with a 1 to 1 stochiometry, the dissociation constant Kd of the complex could be defined as Kd = [PABPN1]×[Hsp70]/[PABPN1-Hsp70]. This constant may be expressed with a double-reciprocal plot equation as a function of the modification of the fluorescence intensities of PABPN1 induced by the presence of increasing amounts of Hsp70:
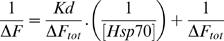
Where Δ*F* is the increase of PABPN1 fluorescence originated by a specific concentration of Hsp70, and Δ*F*_tot_ the maximum increase of PABPN1 fluorescence intensity induced by a saturation concentration of Hsp70 corrected by taking into account the fluorescence modifications induced by the solvent and the added Hsp70 protein alone [51].

### Molecular dynamics simulation of N-terminal PABPN1 peptides

All simulations were performed with the GROMACS package and the all atom GROMOS96 force field [24]. The models used in the simulation were the N-terminal peptides of wild-type PABPN1 protein and extended variants with 3, 5 and 7 Ala extensions as they appeared in OPMD disease. Sequences of each analyzed peptide were for the wild-type PABPN1 N-terminal peptide: M-(A)_10_-GAAGGRG, for 3-Ala expanded: M-(A)_13_-GAAGGRG, for the 5-Ala expanded: M-(A)_15_-GAAGGRG and for the 7-Ala expanded: M-(A)_17_-GAAGGRG. All the simulations were started with fully extended conformations of the peptides with a protonation of side chain consistent with a pH = 7. Peptides were solvated in a water box of 70×70×70 Å and a density of 1 g/cm^3^. The solvated models were energy minimized by conjugated gradient for 1000 steps to eliminate steric clashes between atoms. All the systems were equilibrated by simulated annealing with slow temperature decreasing from 2500 K to 300 K over 1000 cycles. Molecular dynamics simulations were then performed over 1000 ps at 300 K and data collected every 1 ps.

### Conformational probing with fluorescence ligands

Conformational analysis was performed using 1-Anilino naphthalene-8-sulfonic acid (1,8-ANS), a probe that detects exposed hydrophobic domains of proteins. Recombinant PABPN1 protein concentration was 5 µM in 25 mM HEPES buffer, pH 7.5, 100 mM NaCl and 1 mM dithiothreitol. 1,8-ANS was added (final concentration 5 µM) to a solution containing the protein or the buffer only (blank). After a 5-minute incubation, fluorescence emission of 1,8-ANS was scanned from 400 to 650 nm at an excitation wavelength of 380 nm, at 25°C. All experiments were performed with a FS920 Steady-state fluorescence spectrophotometer from Edinburgh Instruments. The band pass was 5 nm for both excitation and emission wavelengths. Means of 5 repetitive scans were recorded for each measurement. The kinetic parameters of the interaction between probe and PABPN1 were estimated using a previously described model [26].

## Supporting Information

Supplementary Material S1 Supplementary material and methods, covering details related with the cloning, expression and purification protocols of Hsp70 chaperone and PABPN1 variants in insect cells, E.coli and Pichia pastoris.
(0.04 MB DOC)
