# Supplementary material for: Hsp70 Chaperones and Type I PRMTs Are Sequestered at Intranuclear Inclusions Caused by Polyalanine Expansions in PABPN1
Source: PLoS One. 2009 Jul 29;4(7):e6418. doi: 10.1371/journal.pone.0006418 (PMC2712759; doi:10.1371/journal.pone.0006418)
Supplement: Supplementary Material S1 — Supplementary material and methods, covering details related with the cloning, expression and purification protocols of Hsp70 chaperone and PABPN1 variants in insect cells, E.coli and Pichia pastoris. (0.04 MB DOC) [file pone.0006418.s001.doc]

**SUPPLEMENTARY MATERIAL AND METHODS**

**Cloning, expression and purification of PABPN1 variants in insect cells**

The normal human PABPN1 cDNA was obtained from the I.M.A.G.E. clone 4672226 and inserted in the pTRE2hyg vector (Clontech) between the *Bam*HI (filled in) and *Nhe*I (ligated to *Xba*I) cloning sites. Expanded PABPN1 cDNA was generated by replacing the first 31 nucleotides of the wild-type coding sequence with a cassette containing 7 extra (GCG) repeats. This synthetic cassette was created by hybridization of two oligonucleotides with the following sequences (5’ to 3’): oligo 1 – GAT CCA TGG CGG CGG CGG CGG CGG CGG CGG CGG CGG CGG CGG CGG CGG CAG CAG CAG; oligo 2 - CTG CTG CTG CCG CCG CCG CCG CCG CCG CCG CCG CCG CCG CCG CCG CCG CCA TG. Hybridization of oligonucleotide 1 to oligonucleotide 2 resulted in a double stranded DNA fragment. The generated cassette consisted of a *Bam*HI cohesive 5’ end followed by the PABPN1 N-terminal coding sequence until the unique *Msp*A1I site at nucleotide 31. This cassette was ligated to *Msp*A1I digested PABPN1 and the reconstituted complete coding sequence was cloned in the *Bam*HI and *Nhe*I (ligated to *Xba*I) sites of the pTRE2hyg vector. The PABPN1 cDNAs were further subcloned into pcDNA3 vector using the *Kpn*I and *Xho*I (ligated to *Sal*I) restriction sites in the polylinker. For baculovirus expression, normal PABPN1 cDNA was subsequently subcloned from the pcDNA3 plasmid through digestion with *Eco*RI and *Xba*I, and inserted into pFastBac™HT A digested with *Eco*RI and *Xba*I; expanded PABPN1 cDNA was isolated through digestion with *Bam*HI and *Xba*I, and inserted into pFastBac™HT B digested with *Bam*HI and *Xba*I. PABPN1 cDNAs were cloned in frame with a hexa-histidine tag present in pFastBac™HT vectors.

Recombinant PABPN1 was produced using the Baculovirus Expression System (Invitrogen). Viral production, viral amplification and protein purification was performed using SF21 cells, according to the manufacturer’s instructions. Briefly, SF21 cells infected with recombinant baculovirus were lysed by sonication in 50mM Tris pH 8.0, 100mM KCl, 5mM 2-mercaptoethanol, 1mM PMSF, protease inhibitors cocktail, 1% NP 40. Lysis was left to occur for 15 minutes on ice and samples were then centrifuged 14000 rpm for 20 minutes. Purification was performed by chromatography on Ni-NTA agarose (QIAGEN, CA). Purified protein was eluted in 0.5ml fractions with 20mM Tris pH 8.0, 100mM KCl, 100mM imidazole, 5mM 2-mercaptoethanol, 10% glycerol, and protein concentration of each fraction was determined with Bradford reagent (Bio-Rad) and/or by SDS-polyacrylamide gel electrophoresis followed by Coomassie staining and gel imaging with bovine serum albumin as a standard. Fractions containing higher protein concentration were pooled together and dialyzed to 20mM Tris pH 8.0, 200 mM KCl and 10% glycerol.

**Cloning, expression and purification of PABPN1 variants in *E. coli***

The cDNA for PABPN1 was amplified by PCR using as a template plasmids pGFP-PABPN1-wt, pGFP-PABPN1-14Ala and pGFP-PABPN1-17Ala (1, 2), using the following primers. Fwd_PABPN1: CACCATGGCGGCGGCGGCGGCGGCG and Rev_PABPN1: GTAAGGGGGAATACCATGATGTCGCTC. Forward oligonucleotide contains the sequence “CACC” in the 5’ terminus that allowed producing PCR products suitable to be cloned in directional TOPO cloning vectors. The amplified PCR products were gel purified and ligated to pET101D vector (Invitrogen). Recombinant plasmids were transformed in *E. coli* Rosetta DE3 (Invitrogen). Cells were growth at 37ºC in 2xTY medium containing 150 µg/ml ampicillin until they reached OD of 0.8 units at 600 nm. Protein expression was induced by addition of 0.2 mM IPTG and cells incubated for 20 hours at 20ºC. For protein purification, cells were resuspended in lysis buffer (50 mM Tris/HCl, 1 M NaCl, 1 mM PMSF, and pH = 8), sonicated and centrifuged at 25,000 x g for 30 minutes. The supernatant was applied to a 5 ml Histrap-FF column (GE-Healthcare), previously equilibrated with buffer A (50 mM Tris/HCl, 0.5 M NaCl, 20 mM Imidazole, pH = 8). The column was washed with buffer A containing 60 mM Imidazole, and the protein bound to the column eluted with a linear gradient of Imidazole. The peak containing recombinant PABPN1 proteins eluted at a Imidazole concentration between 200 and 250 mM. The fractions containing PABPN1 recombinant proteins were pooled, combined and applied to a Superdex 75 10/300 GL (GE-Healthcare) column equilibrated with a buffer containing 50 mM HEPES, 100 mM NaCl, 5 % glycerol, pH = 7.5. Fractions containing protein were pooled and concentrated by ultrafiltration to approximately 1 mg/ml, and stored at -80 ºC until use. Purity of the eluted proteins was tested in all the steps by SDS-PAGE and western blot.

**Cloning, expression and purification of Hsp70 chaperone in *Pichia pastoris.***

Hsp70A cDNA was obtained from I.M.A.G.E. clone 4123837 and amplified by PCR using the following primers: Fwd_Hsp70A : AAAAGAATTCATGGCCAAAGCCGCGGCGATC and Rev_Hsp70A : AAAATCTAGATCAGTGATGGTGATGGTGATGATCCACCTCCTCAATGGTAGG. The primers contained a *Eco*RI (Fwd_Hsp70A primer) and a *Xba*I (Rev_Hsp70A primer) restriction sites to facilitate the cloning of the amplified DNA fragment in the pGAPZα-A vector for protein expression in *P. pastoris*. The Rev_Hsp70A primer also contains a hexa-histidine tag to facilitate further purification of the encoded protein, and a stop codon. The amplified PCR fragments were gel purified, digested with *Eco*RI and *Xba*I enzymes, and ligated with pGAPZα-A vector previously linearized with the same restriction enzymes. Resulting expression vector was transformed in *P. pastoris* X-33 by electroporation and transformants were selected by increasing zeozin concentrations up to 1.5 mg/ml in YPDS medium. Presence of gene encoding Hsp70A was verified by colony PCR using genomic DNA from the transformants as a template. For protein expression, selected transformants of *P. pastoris* were growth at 30ºC in YPD medium for 48 hours. Cells were harvested by centrifugation and the supernatant was collected. Proteins in the extracellular medium were concentrated by precipitation with 75% w/v of ammonium sulfate, and recovered by centrifugation. The protein pellet was resuspended in PBS buffer and desalted by passing thought a PD-10 desalting column (GE-Healthcare). Hsp70A protein was purified from this sample by Ni-affinity chromatography as described for the proteins expressed in *E. coli*.

**REFERENCES**

1. Calado, A., Kutay, U., Kuhn, U., Wahle, E. and Carmo-Fonseca, M. (2000) Deciphering the cellular pathway for transport of poly(A)-binding protein II. *Rna,* **6,** 245-56.

2. Tavanez, J.P., Calado, P., Braga, J., Lafarga, M. and Carmo-Fonseca, M. (2005) In vivo aggregation properties of the nuclear poly(A)-binding protein PABPN1. *Rna,* **11,** 752-62.
